# Supplementary material for: Prevalence and Correlates of Anabolic–Androgenic Steroid Use in Australian Adolescents
Source: Nutrients. 2025 Mar 11;17(6):980. doi: 10.3390/nu17060980 (PMC11945638; doi:10.3390/nu17060980)
Supplement: Supplementary file 1 [file nutrients-17-00980-s001.zip › nutrients-3507966-supplementary.pdf]

Sensitivity Analysis

See Table S1 below for the demographic distribution of Androgenic-Anabolic Steroid use by use of prescription.

Table S1

Demographics of Androgenic-Anabolic Steroid (AAS) Use by Prescription

		Steroid Use			
		No Prescription		Prescription	
		n	%	*N*	%
Biological Sex					
Male		27	84.4	15	79
Female		5	15.6	4	21
Parents born overseas					
Yes		12	41.4	4	23.5
No		17	58.6	13	76.5
Aboriginal or Torres Strait Islander					
Yes		2	6.5	9	50
No		29	93.5	9	50
Same sex attracted or questioning					
Yes		10	31.2	6	31.6
No		22	68.8	13	68.4
	Range	*M*	*SD*	*M*	*SD*
Age	11-19	15.1	1.4	14.3	1.5
BMI percentile	0 - 99.5	66.7	25.6	56.1	35.1

Chi-Square analysis was utilised to investigate whether there was any significant difference in categorical demographic variables, and *t*-tests for continuous variables, across prescribed and non-prescribed AAS use. Only one significant effect emerged - a large effect ($V = .50$) for Indigenous status. A significantly higher proportion of adolescents identifying as Aboriginal or Torres Strait Islander was observed among participants who used prescribed AAS rather than non-prescribed, $\chi 2$ ($df = 1, N = 49$) = 12.41, $p < .001$. No other significant differences were observed between prescribed vs non-prescribed AAS users, including for biological sex, (χ^2 ($df = 1, N = 51$) = 0.24, $p = .62$), same sex attraction (χ^2 ($df = 1, N = 51$) = 0.001, $p = .98$),

migrant status (χ^2 (df = 11, N = 46) = 1.51, p = .22), age (t(44) = -1.88, p = .07), or BMI percentile (t(19.37) = -0.96, p = .35).

Considering the overall similarity in demographic characteristics across prescribed and non-prescribed AAS users, it was determined to combine the groups for the purposes of increased statistical power.
